# Evaluation of Cherenkov-induced photodynamic therapy of biohybrid photosensitive graphene oxide nanoplatform in cervical cancer cells

**DOI:** 10.22038/ijbms.2025.86535.18694

**Published:** 2025

**Authors:** Mona Alikhanzadeh, Armin Imanparast, Elham Einafshar, Hamid Gholamhosseinian, Ameneh Sazgarnia

**Affiliations:** 1 Medical Physics Research Center, Basic Sciences Research Institute, Mashhad University of Medical Sciences, Mashhad, Iran; 2 Department of Medical Physics, Faculty of Medicine, Mashhad University of Medical Sciences, Mashhad, Iran; 3 Pharmacological Research Center of Medicinal Plants, Mashhad University of Medical Sciences, Mashhad, Iran; 4 Department of Pharmacology, Faculty of Medicine, Mashhad University of Medical Sciences, Mashhad, Iran

**Keywords:** Albumin, Cervical cancer, Cherenkov radiation, Graphene oxide, Photodynamic therapy

## Abstract

**Objective(s):**

During radiotherapy, weak photons of Cherenkov radiation are generated, which can cause a relative increase in tumor resistance and cause errors in the radiotherapy treatment planning process. In this study, we used a photosensitive biohybrid graphene oxide nanostructure (GO-BSA-CTAB-PpIX) to maximize the absorption of Cherenkov photons in a broader range of emission wavelengths in order to create the induced photodynamic effect resulting from Cherenkov radiation.

**Materials and Methods:**

TIn the first stage, after the synthesis and surface activation of the graphene oxide nanostructure by EDC, NHS, and albumin, its conjugation process with PpIX was performed. In the second step, it was characterized using an ultraviolet-visible spectrophotometer, dynamic light scattering, Fourier transform infrared spectroscopy, X-ray energy diffraction spectroscopy, and field emission scanning electron microscopy. In the third step, nanodosimetry was performed to prove the Cherenkov-induced photodynamic phenomenon. Finally,* in vitro* cellular studies were performed on the HeLa cell line (cervical cancer). The survival rate of different groups was measured using multiple MTT tests (24 to 72 hr), and the final results were statistically analyzed using GraphPad Prism software.

**Results:**

The results show that the GO-BSA-CTAB-PpIX biohybrid nanostructure can be capable of generating various types of free radicals resulting from photodynamic therapy. This nanostructure inhibits cell growth by disrupting the cellular repair process through natural Cherenkov radiation during radiotherapy and creating an associated photodynamic effect (*P*<0.05).

**Conclusion:**

Cherenkov radiation-based photodynamic therapy is a promising approach to address the challenges of photodynamic therapy and radiotherapy for cervical cancer.

## Introduction

Cervical cancer is the fourth most common cancer and the third cause of death in Africa and South Asia (1). According to the statistics of the World Health Organization, in 2022, about 660,000 new cases of this disease will be diagnosed worldwide, which will lead to the death of 350,000 people (2). Human papillomavirus (HPV) is known to be the main cause of cervical cancer. This virus, which has 15 different genotypes, plays an essential role in the occurrence of two major types of squamous cell carcinoma and adenocarcinoma, which are mainly caused by HPV genotypes 16 and 18 (3, 4). Warning signs of cervical cancer include abnormal vaginal bleeding, pain during intercourse, pain in the back or pelvis, unusual vaginal discharge, blood in the urine, and swelling of one or both legs (4).

Photodynamic therapy is a non-invasive treatment method caused by photochemical reactions between light, photosensitizer (PS), and local oxygen of the tumor. Today, PDT has brought promising results in dermatology, urology, oncology, gynecological diseases, and the treatment of resistant bacterial and microbial infections (5). In this treatment, after the selective accumulation of the photosensitizer in the pathological tissue, the photosensitizer is activated through the absorption of light with the appropriate wavelength. Finally, the direct death of the tumor (necrosis, necroptosis, or apoptosis) occurs. Following PDT, by releasing cytokines and exosomes from PDT-treated tumor cells, the tumor cells are invaded by immune cells, resulting in anti-tumor immune responses and a mesenchymal-to-epithelial tumor phenotype transition, which is useful in controlling tumor cell invasion (5-7).

Due to the limited light penetration depth in tumor tissues, PDT has been used clinically to date only for managing skin diseases and epithelial tumors (8). Since most of the photosensitizers used clinically are activated by UV and visible light at 400–670 nm, when these waves interact with tissue, due to the high absorption and scattering that occurs to them, it is usually between 0.5 and 2.5 millimeters can penetrate healthy tissues, depending on the wavelength. Red and near-infrared light photons with an output spectrum of 600–1200 nm can penetrate healthy tissue to a depth of about 1 cm because blood and skin surface components do not absorb them. However, the main photosensitizers stimulated by infrared wavelengths (e.g., indocyanine green (ICG), etc.) have lower ROS production efficiency than those stimulated by UV waves and visible light. Therefore, limited optical penetration depth is still challenging (7, 9).

One attractive approach to overcome the challenges of limited light penetration depth in photodynamic therapy is to use an intrabody molecular light source instead of an exogenous light source. One of these methods is using Cherenkov radiation as an alternative *in vivo* light source (7). When a charged particle moves at a speed greater than the speed of light in a dielectric medium, it emits continuous radiation, which is a byproduct of nuclear medicine and radiotherapy (6–18 MV), by locally polarizing the molecules around the charged particle and then returning to the steady state (10-14). Cherenkov radiation photons have an emission spectrum in the wavelength range of 250–600 nm, which can excite many photosensitizers in this range (10). Considering the key potentials of CR as a local source of light in deep tissue, it can be used to develop a new method of photodynamic therapy independent of an external source for better penetration depth. 

This study aims to design an albumin-based photosensitive hybrid nanostructure (GBCP: GO-BSA-CTAB-PpIX) in order to maximally absorb the emitted photons from Cherenkov radiation in a wider wavelength range and increase the efficiency of free radical conversion caused by PDT on cervical cancer cells.

## Materials and Methods

***Synthesis and characterization of nanostructures***

*Synthesis of graphene oxide nanostructure*

The modified Hummer method is used for the synthesis of graphene oxide. 500 mg of graphite powder is oxidized in the presence of 500 mg of sodium nitrate and 23 ml of concentrated sulfuric acid. The sample container is cooled in an ice bath, and then potassium permanganate (300 mg) is slowly added to prevent the temperature from rising and causing an explosion. After stirring for two hours at 35 °C in a water bath, we add 140 ml of deionized water to dilute the solution. Next, 3 ml of hydrogen peroxide and 250 ml of 10% hydrochloric acid are added in the next step, and finally, the sample is centrifuged for 20 min at 14000 rpm. Finally, the resulting sample is washed with deionized water until a neutral pH is reached and collected for the next steps (15).

*Activation and surface modification of GO nanostructure*

First, 2 mg of graphene oxide obtained from the previous step was sonicated in 1 cc of deionized water for 30 min. Next, 0.4 mg of EDC was added to it after 45 min, and 1.2 mg of NHS was added to activate the carbonyl groups on the surface of graphene oxide. After about 10 min, the activated graphene oxide obtained from the previous step was combined with 0.019 mg of CTAB and 0.042 mg of bovine serum albumin and stirred for two days on a magnetic stirrer at 1200 rpm to create non-covalent connections.

*Conjugating PpIX to nanostructures*

The surface-modified graphene oxide was then combined with 0.32 mg of PpIX, and the sample was stirred for another day on a magnetic stirrer at 1200 rpm. Finally, the synthetic sample was centrifuged to remove EDC and NHS, which are soluble in water and act as catalysts and activators, along with free substances not attached to graphene oxide.

***Physical and optical characterization of nanostructure***

To perform physical and optical characterization of the nanostructure, ultraviolet-visible spectrophotometer, dynamic light scattering (DLS), Fourier transform infrared spectroscopy (FTIR), X-ray energy diffraction spectroscopy (EDX), and field emission scanning electron microscopy (FESEM) were performed. 

*Ultraviolet-visible spectrophotometer*

An ultraviolet-visible spectrophotometer is an analytical instrument used to measure the absorption or transmission of light in the ultraviolet and visible regions of the electromagnetic spectrum. This study used a UV-Vis spectrophotometer (UNICO UV-2100, made in the USA) in the range of 200–800 nm (1 cm cuvettes) to measure the spectrum of hybrid nanoparticles. 

*Determination of the size of nanoparticles*

The technique of dynamic light scattering, or dynamic light scattering, is one of the suitable methods for determining the size distribution of nanoparticles. In this method, the size distribution of nanoparticles in a solution can be determined from the Brownian motion of nanoparticles in the liquid phase. The speed of the Brownian movement of nanoparticles is related to their size (Stoke-Einstein equation), so the Brownian movement of larger nanoparticles is slower than that of smaller nanoparticles. This study used a particle size analyzer (Nano-ZS, Malvern, UK) to determine the hydrodynamic diameter and zeta potential of GO-BSA-CTAB-PpIX nanostructures.

*Fourier transform infrared spectroscopy (FTIR)*

FTIR analysis is one of the widely used analyses in identifying compounds and bonds in organic and inorganic substances, and it is one of the subsets of spectroscopic analyses (16). By studying the infrared spectrum of the device’s output, chemical bonds, molecular interactions, and the type of functional groups of materials can be identified. This study used a Thermo Nicolet FTIR device (AVATAR 370 FT-IR) to study the bonds in the nanostructure.

*Structural analysis using electron microscopy*

A field emission scanning electron microscope (FE-SEM) is an electron microscope that is primarily used to evaluate the morphological and topological features of nanostructures. In FE-SEM, electrons are ejected from the cathode under the influence of a powerful electric field; the emitted electrons are focused through electron lenses to create an electron beam that is focused on the sample; the electron beam is scanned over the sample surface, and the electrons secondary emissions are collected from the surface of the sample. These signals are converted into an image that provides information about the structure and morphology of the sample. In this study, the TESCAN FEG SEM MIRA3 LMU FE-SEM device was used to investigate the morphology of the final nanostructure.

***Nanodosimetry studies***

*Design of the phantom for registration of Cherenkov radiation*

**Figure 1:**
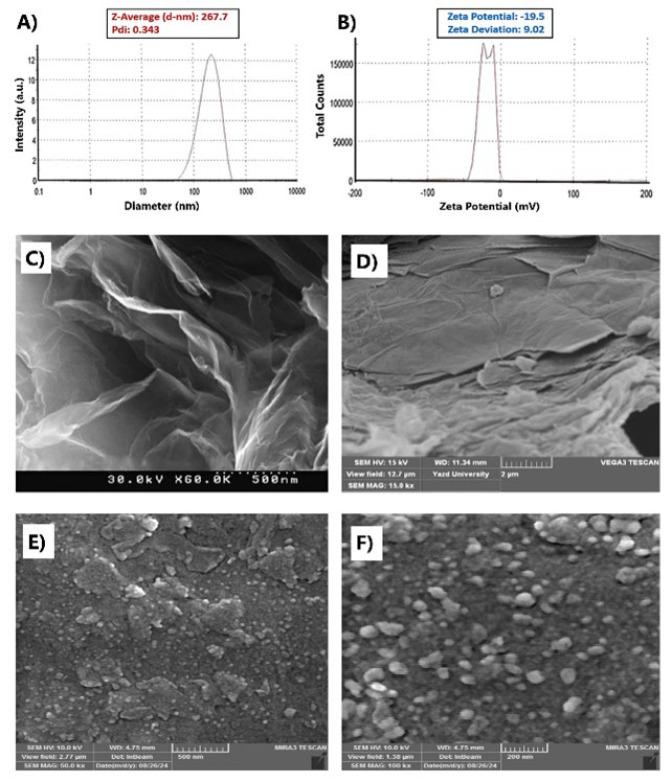
Morphological and structural results of GO-BSA-CTAB-PpIX (GBCP) nanoparticles

In the current research, to conduct water studies and record the spectrum of Cherenkov radiation emitted from water with the help of 3 mm plexiglass plates, a water pond with dimensions of 20 x 20 and a height of 5 cm and considering a hole with a diameter of 1 cm was designed and built to place the spectrometer probe according to Figure 1.

*Recording the emission spectrum of Cherenkov radiation caused by water*

Since Cherenkov radiation is a product of the high speed of charged particles in the dielectric medium, the basin made of Plexiglas plates was filled with distilled water to evaluate the existence of Cherenkov radiation. Then, the radiation was carried out using the Elekta Compact linear accelerator with the radiation position Anterior-posterior, energy 6 mV, dose 200 cGy; the emission spectrum of water was recorded with the help of a spectrometer probe with a collection time of 10 milliseconds, and an average resolution of 1 nm.

*Cherenkov radiation recording nanodosimetry*

In this study, in order to prove the presence of Cherenkov radiation, optical dosimetry of GO-BSA-CTAB-PpIX nanostructure was performed using diphenylisobenzofuran-methylene blue (MB-DPBF) combination in different radiation groups. X-ray radiation was performed using an Elekta Compact linear accelerator with an anterior-posterior irradiation position, energy 6 mV, dose 200 cGy, and using a spectrophotometer, the UV-visible curve of the combined structure of GO-BSA-CTAB-PpIX with MB-DPBF was evaluated.

DPBF material is a single oxygen dosimeter. DPBF has an absorption spectrum in the visible range (λ_max_ = 490 nm) and a fluorescence property that absorbs light with a wavelength of about 410 nm and emits a bright bluish fluorescence. Also, after reacting with singlet oxygen, this chemical probe breaks the p-system of isobenzofuran. It causes the product to be unable to absorb or emit visible light and, as a result, forms o-dibenzoyl benzene. Therefore, the decrease in absorbance or the intensity of the absorption peak or fluorescence emission peak of DPBF indicates singlet oxygen scavenging.

Also, methylene blue (MB) is a natural edible color with two absorption peaks at 610 and 660 nm wavelengths. This material has exceptional optical properties that have made it an exciting factor in diagnostic research and medical treatment. MB undergoes the radio bleaching/photobleaching phenomenon under the influence of free radicals (especially superoxide radicals) caused by radiation or photodynamic therapy. Photobleaching of MB leads to the reduction of its absorption peak at the wavelength of 660 nm, which can be used as a useful dosimetric index.

***In vitro studies***

*Investigating the toxicity of nanostructures*

The HeLa cell line is one of the first and most important cell lines obtained from the cervical tumor tissue of Henrietta Lacks. In this study, the necessary investigations have been done on the HeLa cell line purchased from Pasteur Institute.

In order to determine the toxicity of the nanostructure based on the IC_50_ index, the toxicity that leads to the death of 50% of cancer cells, 10,000 cells were planted in each well of a 96-well plate. After observing the cell density of 80% on the bottom of the cell plate, the GO nanostructure BSA-CTAB-PpIX with different concentration ratios (Table 1) was incubated with cells for 24 hr. Finally, 20 ml of MTT solution and 100 ml of culture medium without FBS were added to the wells, and after wrapping the plate in aluminum foil, it was placed in the incubator. After four hours, MTT was drained, and by adding 200 ml of DMSO and homogenizing with a shaker, an ELISA device was used to read MTT.

*In vitro studies of PDT caused by Cherenkov radiation*

In order to perform *in vitro* studies, 3×10^4^ cells were seeded in each well of the 24-well plate (3 control plates and three radiation plates). After observing the confluency of 80%, GO-BSA-CTAB-PpIX nanostructure at the optimal concentration (determined after toxicity studies) was incubated with cells for 24 hr. After 24 hr, washing was performed with phosphate-buffered saline (PBS), and a culture medium containing 3% FBS was added to each cell well. Then, the cells were irradiated using an Elekta Compact linear accelerator with an energy of 6 MEV (dose of 200 cGy) at a buildup depth of 5 cm. Finally, a culture medium containing 17% FBS was added to the previous medium and placed in the incubator. The MTT method described in the previous section was used to analyze cell survival. MTT assay was performed 24, 48, and 72 hr after treatment.

*Evaluation indices*

In this study, three indices were used to better compare the results. These indices are:

a) **Cherenkov radiation index (CRI):** This index is defined as the ratio of cell death (CD) induced by photodynamic therapy induced by Cherenkov/radiation therapy due to the presence of GBCP nanoparticles (GBCP-RTCR-PDT) to cell death resulting from Cherenkov/radiation therapy (RCTR).

$\mathrm{CRI}=\frac{\mathrm{CD}(\mathrm{GBCP}-\mathrm{RTCR}-\mathrm{PDT})}{\mathrm{CD}(\mathrm{RTCR})}$

b) **Radiation index (RI):** This index is defined as the ratio of cell death (CD) induced by radiation therapy due to the presence of GBCP nanoparticles (GBCP-RTCR-PDT) to cell death. Cell death resulting from radiation therapy (TR).

$\mathrm{RI}=\frac{\mathrm{CD}(\mathrm{GBCP}-\mathrm{RT})}{\mathrm{CD}(\mathrm{RT})}$

c) **Therapeutic efficiency (TE): **This index is the ratio between CRI and RI, which indicates the efficiency of the enhanced treatment due to the presence of induced photodynamic therapy caused by Cherenkov radiation.

$\mathrm{TE}=\frac{\mathrm{CRI}}{\mathrm{RT}}$

***Statistical analysis***

Statistical analysis was performed using GraphPad Prism 9.0 statistical software. All experiments were repeated at least three times, and all results are presented as mean ± standard deviation (SD). Different tests can be used to check the normality of data in GraphPad software. One of the common tests to check the normality of data is the Kolmogorov-Smirnov test. This test allows for comparing data distribution with normal distribution using *P*-value. If the *P-*value is greater than the specified significance level (usually 0.05), it can be concluded that the data follows a normal distribution (*: *P*<0.05, **: *P*<0.01, and ***: *P*<0.001).

## Results

### Results of physical and morphological analysis

*Results of size analysis and FESEM of GO-BSA-CTAB-PpIX*

The results of DLS analysis in Figure 1 A-B for GO-BSA-CTAB-PpIX nanoparticles show that the average hydrodynamic diameter (z-average) is 267 nm. This size is relatively large, which can be due to the presence of proteins on the nanoparticle surface. The results obtained in this study are similar to those of Liu *et al*. (17). Regarding the size of GO, since most graphene-based materials are not spherical particles, the diameters derived from the model cannot describe their actual size, and the nominal size distribution of nanoparticles can be estimated according to the SEM results (17). Also, the PDI of the system is 0.343, which indicates the optimal size distribution of nanoparticles in the sample. The closer the PDI value is to zero, the more uniform the particle size distribution is. However, protein-containing nanostructures generally do not have an ideal dispersion index. On the other hand, the zeta potential of the system is -19.5 mV, which indicates the surface charge of the particles and is due to the ionization of carboxylic acid and phenolic hydroxyl groups on the GO nanoparticle sheets. According to Figure 1D, graphene oxide is a flat sheet and completely planar, but due to the oxidation process of graphene, wrinkles can be seen in the pictures of graphene oxide. In addition, the negative surface charges will lead to greater stability of the GO nanoparticle colloidal solution due to the electrostatic repulsion between them (18). 

Due to the two-dimensional planar structure of graphene oxide, its morphology is evaluated by field emission scanning electron microscope (FESEM). In Figure 1(E-F), the micrographs of GO-BSA clearly show the change in the structural morphology of GO. A wrinkled lamellar structure is observed after conjugation, indicating covalent interaction. The observed morphology of GO-BSA can be attributed to the amide groups of BSA and the carboxyl groups of GO, as well as the hydrogen bonds between the hydroxyl and amino groups of BSA and the oxygen groups of GO (19). The FE-SEM images of GBCP nanoparticles should reveal well-dispersed, spherical, or sheet-like nanostructures anchored on the GO surface. The absence of large aggregates or irregular clusters confirms the success of the composite fabrication process. The BSA (bovine serum albumin) and CTAB (cetyltrimethylammonium bromide) likely act as stabilizers. BSA provides steric hindrance and biocompatibility, and CTAB enhances electrostatic stabilization via its cationic charge.

Together, these components mitigate van der Waals forces and π-π stacking between GO layers, ensuring a uniform distribution of PpIX (the photosensitizer) across the composite.

*Results of spectrophotometric analysis results of GO-BSA-CTAB-PpIX*

**Figure 2:**
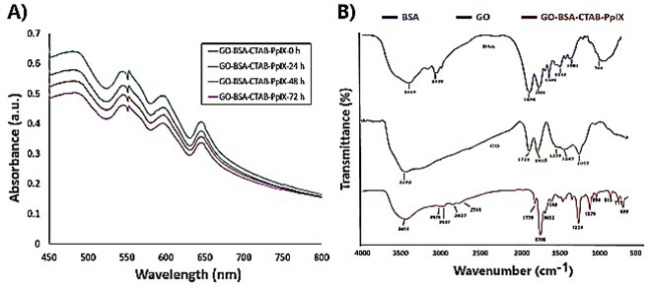
Spectrometric analysis of nanostructures

Among different proteins, serum albumins are of special importance because they can bind to different compounds and transport them through blood flow in the organism. In addition, the high affinity of albumins to malignant tissues makes them a promising drug delivery system. On the other hand, the binding of photosensitizers to albumins changes their photophysical properties, which can affect their efficiency in photomedical applications. Environmental characteristics, such as pH, can affect the interaction between PS and albumins due to changes in their charge states and/or compositions. Therefore, the effect of pH should be considered. As seen in Figure 2-A, the time-dependent changes in the absorption spectrum of the GO-BSA-CTAB-PpIX hybrid nanostructure indicate that the intensity and position of the absorption peaks undergo very slight changes after 72 hr, indicating the relatively good stability of this nanostructure at 4 °C temperature. The prominent peaks in the wavelength range of 500 to 650 nm are attributed to protoporphyrin-9. These peaks are related to electron transfers in the protoporphyrin structure. A slight shift in the position of the peaks is also observed, possibly due to changes in the surrounding environment of protoporphyrin molecules and intermolecular interactions.

*Results of FTIR results*

Fourier transform infrared spectroscopy (FTIR) was used to confirm the synthetic particles’ structure, surface composition, and functional groups (Figure 2-B). FTIR spectra of BSA, GO, GO-BSA-PpIX, and GO-BSA-CTAB-PpIX compounds were compared. BSA showed the same FTIR spectrum as other protein substances. The prominent band at 1656 cm^-1^, known as the amide I band, represents the strong carbonyl group in BSA. This is expected in a protein with a significant proportion of the α-helical structure. The band at 1534 cm^-1^ was attributed to the amide II band, a combination of C-N and N-H stretching and bending in secondary amides. The presence of primary amines was associated with the band at 3318 cm^-1^. In addition, the band at 2959 cm^-1^ indicates a C-H vibration, while the broadband at 701 cm^-1^ indicates the presence of -NH2 and -NH stretching (20). The FTIR spectrum of the GO sample also matched well with the studies (21, 22). The O-H stretching bond, indicating the presence of hydroxyl groups in GO, was observed in the high-frequency region with a peak in the range of 3690 cm^-1^ to 3100 cm^-1^. Absorption bands at 1721 cm^-1^ and 1339 cm^-1^ were also identified, which are related to the stretching vibrations of carboxyl and carbonyl groups. The peak at about 1615 cm^-1^ represents the sp^2^ (C-C) carbon skeleton network, while the C-O stretching vibrational signals in the epoxy groups were detected at 1055 cm^-1^ and 1247 cm^-1^ (23, 24).

In the final combination of GO-BSA-PpIX and GO-BSA-CTAB-PpIX, all distinct vibrational modes associated with BSA and GO were observed. The C-O group signal at 1615 cm^-1^ in PpIX suggests a unique arrangement in which PPIX aggregates with another PPIX molecule through π-π interactions and hydrogen bonding between their carboxylic groups (25). However, in the PPIX-BSA-GO conjugate, this signal shifts to 1652 cm^-1^, indicating that the hydrogen bonds are broken as PpIX dissociates after conjugation to graphene oxide. Moreover, the characteristic vibrational modes of CTAB, such as strong absorptions at 2937 cm^-1^ and 2827 cm^-1^ due to the C-H stretching vibration of the methyl and methylene groups of CTAB, are clearly detected (26, 27).

### Results of Cherenkov radiation dosimetry in water environment

*Results of registration of Cherenkov radiation*

**Figure 3:**
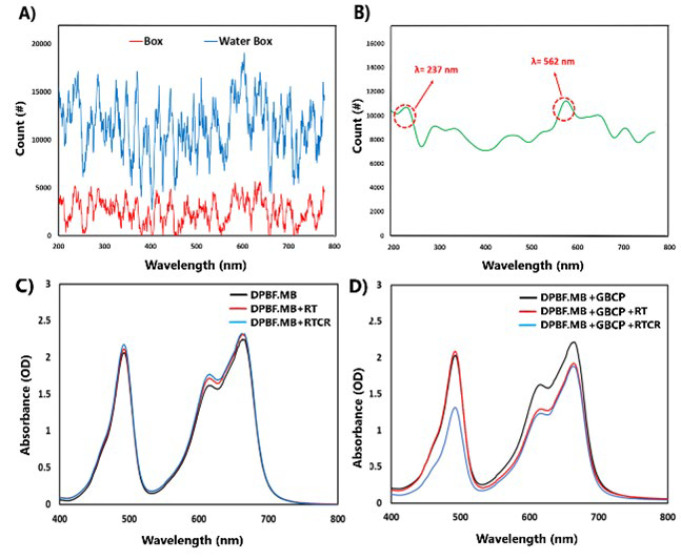
Nanodosimteric analysis data

The results of the spectrometer in Figure 3-A show that the fluorescence intensity of the box containing water is higher than that of the empty box. The increase in the radiation intensity in the presence of water indicates the production of Cherenkov radiation. The wavelength range recorded from the water-filled box is in the visible and ultraviolet ranges, consistent with the expected range for Cherenkov radiation. The different peaks in the spectrum may be due to various factors, such as the type of X-ray used, the energy of the electrons produced, and the complex interactions between the particles and the medium. The differential results (Figure 3-B) clearly show that the light photons resulting from Cherenkov radiation exist at two wavelengths, 562 nm and 237 nm.

*Results of singlet oxygen and hydrogen peroxide dosimetry*

MB, as a photosensitizer, produces ^1^O_2_ when exposed to light. This substance also undergoes bleaching in case of free radical production (especially H_2_O_2_) caused by irradiation. So, in certain conditions, it can play a role as a free radical dosimeter. Also, DPBF is a substance known as a ^1^O_2 _chemical dosimeter. By using the DPBF-MB combined dosimeter solution, it is possible to check which phenomenon of free radical production occurs during the two processes of X-ray irradiation (RT) and photodynamics induced by Cherenkov radiation (CR-PDT) on the GO-BSA-CTAB-PpIX hybrid nanostructure. (^1^O_2 _or ROS) is dominant.

Before investigating the potential of free radical production by GO-BSA-CTAB-PpIX hybrid nanostructure (abbreviated as GBCP), the radio bleaching effect of DPBF-MB solution under X-ray and Cherenkov photons was investigated. The results of Figure 3-C show that the combined DPBF-MB solution is not so affected by X-ray radiation and Cherenkov photons, so it can be claimed that it is a stable structure.

The combined solution of DPBF+MB+GBCP was exposed to X-ray radiation (RT) and X-ray radiation plus Cherenkov photons (RT+Cherenkov). The results of Figure 3-D show that in the RT radiation group, the peak attributed to methylene blue at the wavelength of 670 nm has undergone bleaching, which is caused by the production of free radicals (mainly hydrogen peroxide type) by GBCP hybrid nanostructure. Also, in the group under RT+Cherenkov radiation, both the absorption peaks of MB and DPBF have bleached, which indicates the production of free radicals (hydrogen peroxide and singlet oxygen) due to the presence of the GBCP hybrid nanostructure, which in a way indicates the occurrence of the CR-PDT phenomenon.

Existing studies show that experimental conditions can influence the interaction of nanoparticles with proteins (such as hemoglobin, albumin, etc.) (28). In our system, (DLS/Zeta Potential) measurements confirm that the stability of the particles is maintained and that the high absorption is due to the high intrinsic optical properties of the structures, not to the formation of aggregates.

### Results of in vitro studies

*Data normality check*

The normality of the data was checked using GraphPad Prism 9 software, as shown in Table 2. The Normal plot and Kolmogorov-Smirnov test results show that all the data follow the normal distribution.

*Results of investigation of GBCP toxicity*

After optimizing the nanostructure in terms of optical and physical properties, the optimal concentration ratio was determined for the GO-BSA-CTAB-PpIX (or GBCP) hybrid nanostructure. These optimal ratios based on µg/ml are:

GO: BSA: CTAB: PpIX = (1:21:95:0.3125) (Formula 1)

**Figure 4:**
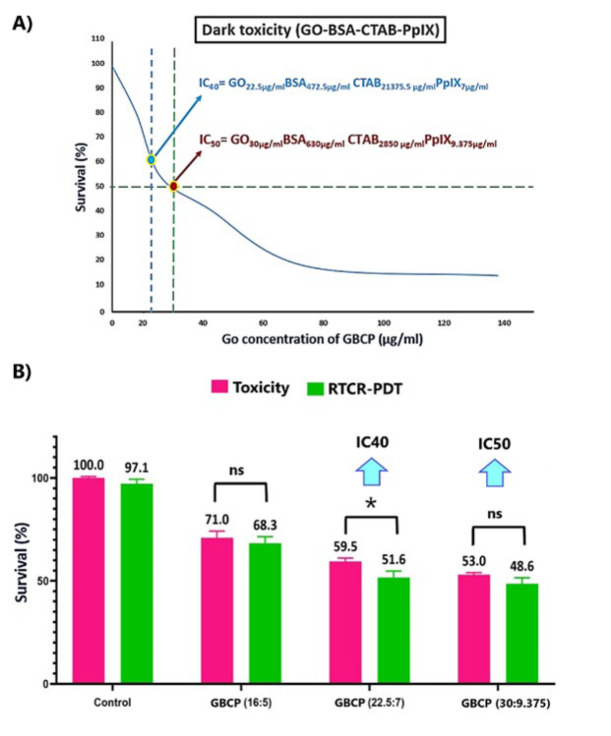
A) Toxicity of GBCP hybrid nanostructure in different concentrations based on GO concentration. B) Results of RTCR-PDT toxicity compared to the toxicity group (without radiation) in the presence of hybrid nanostructure (GBCP: GO-BSA-CTAB-PpIX) with 6 MeV energy and 200 cGy dose on cervical cancer cells (Hela)

This formula represents the most optimal normalized concentration ratio between the nanostructure components that can maintain its optical/physical/chemical stability in an aqueous solution longer than other concentrations. Only the concentrations of the two main components, GO and PpIX, are expressed for easier addressing. For example, wherever GBCP_22.5/7 _is written, it means a concentration of 22.5 μg/ml GO versus a 7 μg/ml PpIX concentration. The concentrations of BSA and CTAB can be calculated based on the ratio information in Formula 1. Based on this, by maintaining the optimal concentration ratio between the different components of the nanostructure, its toxicity on the HeLa cell line was investigated. The results of Figure 4-A show that the IC_50_ and IC_60_ indices of the hybrid nanostructure are GBCP_30/9.375 _and GBCP_22.5/7_, respectively. The study was continued with the IC_60_ concentration. 

*Results of investigation of X-ray and Cherenkov radiation*

The results of Figure 4-B show the comparison of the cell viability caused by the combination of radiation therapy and Cherenkov radiation (RTCR) with 6 MeV energy and 200 cGy dose on cervical cancer cells incubated with different concentrations of GBCP hybrid nanostructures after 24 hr compared to the control group. In groups where RTCR occurs in the presence of GBCP nanostructure, we face the induced photodynamic phenomenon caused by the combination of radiation therapy and Cherenkov radiation, which we call GBCP-RTCR-PDT. According to the results, it can be concluded that choosing IC_60_ compared to IC_50_ can provide better therapeutic efficiency compared to the corresponding toxicity group.

*Results of comparison of the effect of GBCP-RT and GBCP-RTCR-PDT under the optimal concentration of nanostructure*

**Figure 5:**
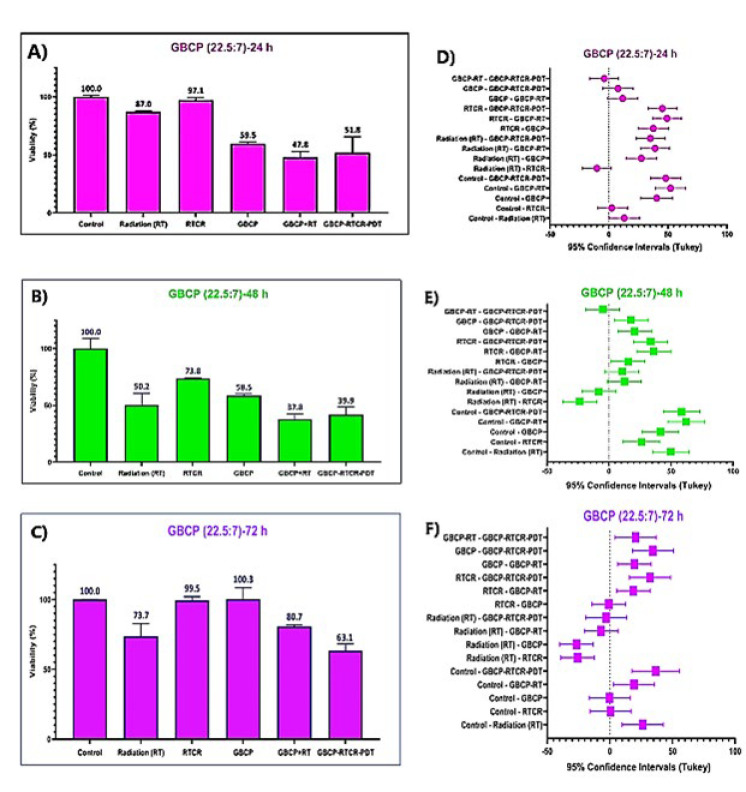
Results of measuring cell survival caused by radiotherapy and Cherenkov radiation in the presence of GBCP hybrid nanostructure at A) 24 hr, B) 48 hr, and C) 72 hr after treatment. The 95% confidence interval (CI) values for different treatment groups after treatment (time intervals of D) 24 hr, E) 48 hr, and F) 72 hr. If the values of any of the data (including the error bars) touch the dotted line, it means that the changes are not significant (*P*<0.05). On the other hand, if any of the data is far from the dotted line, the results are significant (*P*<0.05). The degree of significance of the results is proportional to the distance of the desired interval from the dotted line. Data are shown as the mean values of five replicates (±) standard deviation.

The results of Figure 5-A show the effect of radiation therapy and Cherenkov radiation with 6 MeV energy and 200 cGy dose on the survival of cervical cancer cells incubated with GBCP_22.5/7 _after 24 hr. The difference between GBCP-RT and GBCP-RTCR-PDT groups is due to the presence/absence of Cherenkov radiation Photons according to the radiation setup, which was explained in detail in the last chapter. The results indicate that the GBCP+RT group has the highest toxicity rate (48% survival). Compared to the corresponding radiation control group (RT group), the survival rate decreased from 87% to 48% (39% difference). In the GBCP-RTCR-PDT group, the survival difference in the control group is 48% (survival 52%). However, the highest difference compared to the corresponding radiation control group (RTCR) is 45%.

Figure 5-B shows the results of the survival test 48 hr after the treatment of cells incubated with GBCP under RT and RTCR radiation. As it is known, the highest rate of cell death belongs to the GBCP+RT group. The GBCP+RTCR-PDT group had an inhibitory effect compared to the RT group and its corresponding control group (RTCR).

Figure 5-C shows the results of cell survival 72 hr after treatment. The proliferation mode was observed in all groups, compared to 24 and 72 hr. Noteworthy in the MTT test is that the group incubated with RTCR had more survival than the group incubated with RT. This behavior has also been observed in multiple MTT assay times. Also, comparing the cell survival results in the GBCP+RTCR-PDT groups with GBCP+RT shows that the GBCP+RTCR-PDT group significantly differs in inhibitory properties. (*P*<0.05).

Figures 5-D to 5-F also show the treatment groups’ 95% confidence interval (CI) values ​​. If the values ​​of any data (including the error bars) intersect the dotted line, it means that the changes are not significant (*P*<0.05). On the other hand, if any data is far from the dotted line, it means that the results are significant (*P*<0.05). The degree of significance of the results is proportional to the distance of the desired interval from the dotted line. Data are shown as the mean values ​​of five replicates (±) standard deviation.

*Results of the time-dependent behavior of the survival curve after radiation*

**Figure 6:**
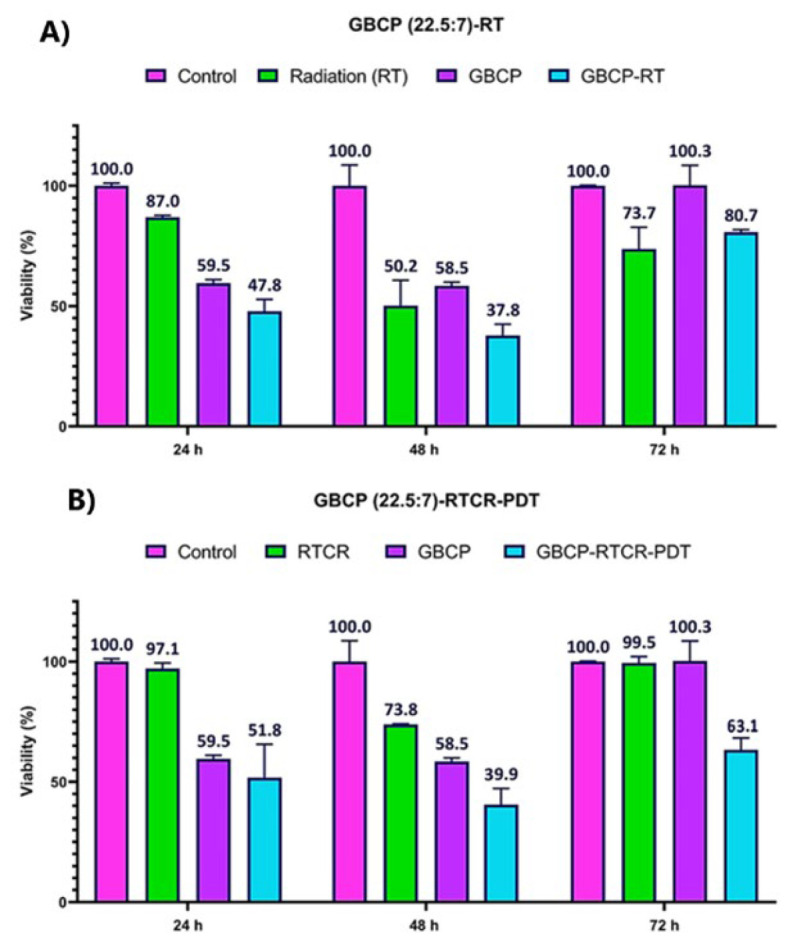
Comparison of changes in cell survival caused by A) GBCP+RT and B) GBCP+RTCR-PDT at 24 hr, 48 hr, and 72 hr after treatment

Figure 6 (A, B) shows the changes in cell survival between 24 and 72 hr after irradiation. As the results show, with the passage of time in the cell division process of the GBCP+RTCR-PDT group, more disturbance is observed than in the GBCP+RT group. Cherenkov’s radiation index (CRI) has larger values ​​than empty radiation therapy efficiency (RI) (TE greater than 1), which indicates the greater effectiveness of the RTCT-PDT process compared to RT (Table 3).

## Discussion

Cherenkov radiation (CR), which has attracted increasing attention in health, is produced through the interaction of charged particles and matter. This radiation can be produced from radionuclides, megavoltage X-ray radiation, cosmic events, and nuclear reactors. Cherenkov radiation has great potential in imaging and therapeutic applications in line with effective cancer management. Cherenkov luminescence imaging systems can be used in fields such as surgery to define tumor margins and in radio-oncology to develop real-time dosimetry techniques and guided imaging during radiation therapy.

Despite the great advantages of Cherenkov radiation, the low efficiency of its emission of Cherenkov photons, and the interference of ambient light, its widespread use has been limited. By taking advantage of nanotechnology, the maximum capacity of CR can be used to improve patient outcomes and develop new treatment techniques.

Carbon-based nanomaterials have attracted much attention in industry, medicine, electronics, and optics due to their high surface-to-volume ratio, rigid and stable structure, and suitable biocompatibility. Graphenes and their derivatives, such as graphene oxide, are among the most important carbon-based structures. These materials, as single-layer carbon materials, facilitate easy modification with biomolecules due to the presence of functional groups such as epoxy, hydroxyl, and carboxylic acid groups attached to them, and as a result, a large cross-sectional area for loading nucleobases, aromatic compounds, and hydrophobic photosensitizers (mainly through π_π stacking). In this way, they prevent the deactivation of photosensitizers before reaching the target, increasing cellular internalization and photodynamic therapy activities due to electron sinking.

Considering the key potentials of CR as a local source of light in deep tissue, it can be used to develop a new method of photodynamic therapy independent of an external source for better penetration depth. In this research, from this CR potential, investigate *in vitro* the effect of photodynamic therapy induced by Cherenkov emission by the hybrid nanostructure of graphene oxide-albumin-surfactant-protoporphyrin (GBCP: GO-BSA-CTAB-PpIX) on It was performed on cervical cancer cells.

Graphene oxide (GO) is a graphene derivative with a two-dimensional atomic layer composed of sp^2^ and sp^3^ carbon with oxygen functionalities such as epoxide, hydroxyl, and carboxylic groups. The hexagonal carbon structures and these functional groups lead to versatile surface chemistry and allow the formation of covalent and non-covalent bonds. The rich surface chemistry makes GO more popular than pristine graphene in biomedical applications such as drug delivery (29). In addition, compared to graphene, which is insoluble in biological solutions and tends to aggregate, GO has excellent water solubility. The free π-electrons in the unmodified regions of GO provide hydrophobic regions suitable for loading hydrophobic drugs through van der Waals forces (30).

Different tissues and cellular compartments of the body have specific pH levels that can stimulate pH-sensitive drug delivery systems (31). In some abnormal physiological conditions such as cancer, inflammation, and infection, significant changes in pH have been identified in diseased areas. For example, in tumor tissue, glycolysis occurs at high speed, and lactic acid accumulates due to the rapid proliferation of tumor cells and lack of nutrients. This leads to a significant decrease in pH in the tumor microenvironment (5.5–6.8) compared to blood and normal tissues (≈7.4), which can act as an endogenous stimulus for acid-sensitive drug delivery systems (31-33). In acidic environments, hydrophobic drug molecules such as doxorubicin undergo protonation, and hence, π-π stacking and hydrophobic interactions with the graphene surface are weakened, leading to drug release with pH changes (34).

Electrostatic interaction, hydrophobic interaction, van der Waals force, and hydrogen bonding are the primary forces that help to form the protein corona. Protein adsorption on the surface of nanomaterials ultimately changes the result and may even cause cytotoxicity (35, 36). The protein corona can increase the size of the nanomaterial or change its charge by surface adsorption, which can also change the biological signal. Structural changes in protein adsorbed on nanomaterials can disrupt protein function and ultimately cause toxicity. To prevent such toxicity, nanoparticles can be coated with a special protein to act as a protective sheath that prevents the absorption of any other plasma protein. This protective protein coating reduces rapid clearance to achieve long-term circulation (35, 36).

Human serum albumin constitutes 52 to 62% of the total blood plasma protein, which indicates its abundance. Bovine serum albumin (BSA) and human serum albumin are structurally similar, so they are used in research (37).

On the other hand, although GO nanoparticles have been widely studied for potential biomedical applications, the potential risk associated with GO interactions in a biological system hinders their biomedical applications. Therefore, there is an urgent need to increase the biocompatibility of GO (38). In this research, GO was coated with an optimal concentration of BSA to increase its biocompatibility. Finally, GO’s connection with BSA was confirmed by FTIR and FE-SEM. The FTIR spectrum of BSA shows characteristic amide bands (N-H, C=O), while GO exhibits typical oxygen-containing groups (C=O, C-O-C, C-OH). The spectrum of the GO-BSA-CTAB-PpIX composite clearly shows the presence of characteristic peaks from all components, including the amide bands of BSA (with observed shifts indicating interaction), oxygen functionalities of GO, and new peaks corresponding to the C-H stretching vibrations from CTAB and characteristic bands of PpIX. Comparing the spectra, the observed shifts and changes in the intensity and position of key bands, particularly the amide I and II bands of BSA and potentially the C=O band of GO, provide strong spectroscopic evidence for favorable interactions (such as hydrogen bonding and electrostatic interactions) between BSA, GO, CTAB, and PpIX. These interactions facilitate the successful incorporation of the components and the formation of the stable composite structure rather than indicating the breakage of core functional groups. This analysis confirms all components’ successful synthesis and interaction within the final composite (39).

Another feature of BSA is its fluorescence properties. Since BSA can lead to the retention of PpIX, PpIX molecules are placed the closest to the amino acid of BSA and can emit its fluorescence. (resulting from the Cherenkov radiation process) are stimulated and produce ROS/^1^O_2_. This phenomenon is the indirect effect of photodynamics induced by Cherenkov radiation.

One key advantage of PpIX compared to other photosensitizers is the presence of various absorption peaks in the ultraviolet and visible regions. In standard photodynamic therapy protocols based on coherent/incoherent light sources for tumors near the skin surface, only one wavelength (630 nm) is usually used, and other wavelengths in blue areas are ignored for PDT purposes due to limited penetration depth. Therefore, the maximum potential of PpIX is not used to obtain the optimal therapeutic efficiency (40).

BHowever, during Cherenkov radiation, although the intensity of Cherenkov light from X-rays is much lower than the coherent/incoherent light used in PDT, Cherenkov light may still produce an acceptable therapeutic response due to several unique properties, including:

a) Sort band effect: Due to the excitation spectrum of PpIX, the absorption efficiency of Cherenkov light is much higher than the absorption efficiency of laser light. For example, the absorption efficiency of 400–405 nm is at least 20–30 times higher than that of 630 nm (40).

b) No attenuation and scattering at the production site: The external laser light irradiated to the tissue to perform PDT is immediately attenuated and scattered so that the light intensity at a depth of 7 mm under the skin reaches about 10% of the surface (initial intensity). However, Cherenkov radiation is less attenuated and scattered since it is generated inside the body and at the tumor-tissue interface (40).

GBCP hybrid nanostructures (GO-BSA-CTAB-PpIX) contain BSA protein. BSA contains aromatic amino acids such as tryptophan, tyrosine, and phenylalanine. These amino acids have benzene rings that absorb ultraviolet light and then return to the base state by emitting light at longer wavelengths (visible region). The energy absorbed by an aromatic amino acid can be transferred to the adjacent amino acids and cause fluorescence emission from them. BSA fluorescence amounts depend on various factors such as solvent type, pH, temperature, protein concentration, and excitation wavelength.

Typically, wavelengths of 280 nm (for tryptophan) and 275 nm (for tyrosine) are used to excite fluorescence in BSA. As the results of the current study show, when the X-ray radiation hits the water phantom, we will see Cherenkov emission photons with different wavelengths in the ultraviolet-visible regions. So, the Cherenkov emission spectrum also has a relative extremum at 275 nm and 280 nm wavelengths. Therefore, it can be interpreted that GBCP hybrid nanostructure under X-ray irradiation has the potential to produce fluorescence. This fluorescence caused by BSA, which is in the visible region, is absorbed by PpIX molecules, which can cause the excitement of this molecule. On the other hand, the presence of various emission relative extrema in other wavelengths caused by Cherenkov emission (especially at the wavelength of 562 nm) can also stimulate the GBCP hybrid nanostructure and produce free radicals. This hypothesis was well confirmed according to MB-DPBF dosimetry results.

IFuture studies could systematically evaluate charge-dependent behavior by synthesizing GBCP variants with tunable zeta potentials (e.g., via alternative surfactants) to dissect its impact on cellular internalization and radiation-activated PDT efficacy.

In gold nanostructures carrying protoporphyrin-IX (PpIX) coated with BSA protein for radiodynamic therapy (Cherenkov radiation-induced photodynamic therapy), the mitochondria are the primary target inside the cell. PpIX naturally accumulates in mitochondria, and when exposed to Cherenkov radiation, it generates reactive oxygen species (ROS) that damage the mitochondrial membrane and activate apoptotic pathways. The BSA coating enhances nanoparticle stability and cellular uptake, facilitating effective delivery of PpIX to mitochondria (41).

As shown in Figure 5 (A:C), at all survival measurement times, the survival of the RTCR group was significantly higher than that of the RT group. The reason for this phenomenon is that under real *in vivo* conditions, Cherenkov radiation is always generated at the tumor and tissue cross-section, but due to the very low light flux, it does not have a significant therapeutic effect. This can probably have two different reasons (42). The following two hypotheses are considered for a more detailed analysis. These two hypotheses are:

A) Pseudo-hormesis hypothesis: proliferation of cancer cells under mild ultraviolet/visible Cherenkov background radiation (43).

B) The cancer stem cell differentiation induction hypothesis involves stimulating the differentiation behavior of cancer stem cells into normal cancer cells under mild ultraviolet/visible Cherenkov background radiation (42).

Hormesis is any biphasic dose response in which low doses of some agents are beneficial and higher doses are harmful. By applying low doses of light (ultraviolet to near-infrared), cancer cells’ proliferative behavior can be stimulated. Considering that during radiation therapy, Cherenkov photons are always produced at the interface between tumor and tissue, and their optical radiation flux is very low, these photons can stimulate the proliferation and resistance mechanisms of cancer cells, which finally appears with the increase of reproduction in the survival curve.

This behavior can be seen in Figure 5 (A:C). Although the GBCP+RT and GBCP+RTCR groups are not significant compared to each other, they are significant compared to their corresponding radiation control groups (RT group for GBCP+RT and RTCR group for GBCP+RTCR) and the GBCP+RTCR group has therapeutic efficiency. It is relatively better (compared to the corresponding radiation control group).

Cancer stem cells (CSCs or tumor-initiating cells) are a small part of tumor cells that can self-renew, multiply, and differentiate into different cell lines. Cancer stem cells in specific microenvironments) are resident. CSCs are composed of different types of cells that maintain life and improve the characteristics of CSCs.

When cancer stem cells are exposed to chemicals or light radiation, they multiply and differentiate into differentiated cancer cells, which can cause the proliferation of cancer cells and tumor growth. Although the volume of the cell mass is increased in this case, its resistance is much lower because normal cancer cells have fewer protective mechanisms than CSCs. Recent studies have shown that the cause of intratumoral heterogeneity in cervical cancer is the presence of stem cells because CSCs usually undergo asymmetric division. Thus, distinct tumor cell populations exhibit different molecular and phenotypic characteristics associated with poor response to chemotherapy and/or radiotherapy and increased risk of lymph node metastasis and pelvic recurrence (44, 45). Recent biomechanical studies suggest that using nanostructures is a new approach to combat cancer called “differentiation-based nanotherapy”. GO nanostructures are non-toxic to normal stem cells and enhance their differentiation. However, these nanostructures can be used directly as a therapeutic to target cancer stem cells, possibly as a differentiation agent. Depending on the tumor location, GO could play a role as a new anticancer drug. In addition, GO could be used as a lavage solution during surgery to clear the tumor removal site or peritoneal cavity (such as ovarian cancer or other peritoneal cancers) of remaining cancer stem cells to prevent tumor recurrence and distant metastasis (46).

Since GO nanoparticles have a size of several tens of nanometers in one dimension and a size of several micrometers in the other two dimensions, they exert their major effects at the cell surface and reduce the activation of several stem cell-related signaling pathways that initiate at the cell surface. As a result, the large size of GO nanostructures is too large for effective cellular uptake (internalization into cells), and these nanostructures do not significantly affect mitochondrial oxidative phosphorylation in this context because they do not target mitochondria. However, studies show that reduced oxygen in mitochondria caused by PDT-mediated ROS production inhibits cancer cell stemness because CSCs have more pronounced anaerobic glycolytic properties than differentiated cancer cells. It has been inferred that under hypoxic conditions, some CSCs preferentially undergo oxidative phosphorylation to survive, maintain stemness, and switch to glycolytic metabolism during differentiation. However, during PDT, a large amount of oxygen is consumed, forcing CSCs to switch from oxidative phosphorylation in the “stem cell” state to anaerobic glycolysis during differentiation, indicating that PDT can effectively control CSC differentiation by affecting mitochondrial function (46, 47).

In this study, as shown in Figures 5-A to 5-C (cytotoxicity after 24, 48, and 72 hr after treatment, respectively), when cells were exposed to low-dose Cherenkov radiation from radiotherapy, the proliferative behavior of cells in all groups was observed compared to the single radiotherapy group. In fact, during radiotherapy, X-rays destroyed a portion of the cancer cells, but Cherenkov radiation caused relative proliferation by differentiating the cancer stem cell population.

As shown in Figure 5-C, 72 hr after treatment, the GBCP+RT group did not report a significant difference compared to its corresponding radiation control group (RT), indicating the repair of radiation damage after 72 hr. However, in the GBCP+RTCR-PDT group, survival after 72 hr was about 36% lower than the corresponding control group (RTCR), indicating that the cell repair system was impaired, which could be due to a decrease in the population of primary cancer stem cells under this type of treatment. Further research is needed to prove this claim. Given that after 72 hr, cell survival increased in all groups compared to 24 and 48 hr, it seems that if the subsequent supplementary treatment is performed between 48 and 72 hr, considering the theory of induction of cancer stem cells into non-stem cancer cells after the first photodynamic treatment session, it can be expected that cell death in the GBCP+RTCR-PDT group will increase significantly.

Cellular signaling pathways play a crucial role in determining the therapeutic outcomes of cancer treatments, including photodynamic therapy (PDT) and radiodynamic therapy (RDT). Among these, the PI3K/AKT pathway is a key regulator of cell survival, proliferation, and resistance to apoptosis, and its modulation by reactive oxygen species (ROS) has been widely reported. As highlighted in previous studies, oxidative stress generated during PDT or RDT can disrupt mitochondrial function, leading to the activation or inhibition of ROS-sensitive pathways such as PI3K/AKT, MAPK/ERK, JNK, and NF-κB. Previous studies discuss these pathways’ involvement in PDT-mediated apoptosis, where MAPK/ERK can influence cell cycle arrest, JNK is associated with stress-induced apoptosis, and NF-κB can either promote survival or contribute to cell death depending on the cellular context. Similarly, findings from related studies suggest that ROS-mediated mechanisms could indirectly downregulate PI3K/AKT, enhancing apoptotic responses. Although this study did not directly investigate signaling pathways, the GBCP nanostructure employed in RDT likely interacts with these pathways through its ROS-mediated effects on mitochondria. These insights underline the therapeutic potential of GBCP in targeting cervical cancer and highlight the need for future studies to explore its molecular mechanisms, particularly its influence on pathways like PI3K/AKT, MAPK/ERK, JNK, and NF-κB (48, 49).

The clinical feasibility of this approach is particularly promising due to its utilization of intrinsic Cherenkov radiation generated during standard radiotherapy. This elegant mechanism naturally bypasses the light penetration limitations inherent in conventional PDT while requiring no additional specialized equipment. The seamless integration potential with existing radiotherapy protocols significantly enhances its translational potential, especially when considering the encouraging preliminary efficacy results we have observed.

RWe have taken proactive measures regarding potential toxicity concerns in the nanoparticle design phase. Our GO-BSA-CTAB-PpIX nanostructure incorporates a BSA coating specifically to enhance biocompatibility, and preliminary cytotoxicity assessments have yielded reassuring results. We recognize that careful optimization of radiation dosing parameters will be crucial to minimize potential toxicity to surrounding healthy tissues while maintaining therapeutic efficacy.

For protocol optimization, we are implementing a multifaceted strategy. This includes systematic evaluation of nanoparticle component ratios to maximize ROS production efficiency, comprehensive assessment of critical treatment parameters such as radiation dose and nanoparticle concentration, and development of tumor-specific targeting mechanisms. Additionally, we are planning detailed pharmacokinetic studies to better understand the *in vivo* behavior of our nanostructures. These optimization efforts will form the foundation of our upcoming preclinical studies as we work toward clinical translation.

## Conclusion

Cherenkov radiation is a type of electromagnetic radiation that is emitted when a charged particle (such as an electron) passes through a dielectric medium (such as distilled water) with a speed greater than the phase speed (speed of light propagation). Cherenkov light is spontaneously produced in tissues exposed to ionizing radiation in a continuous spectrum from the near ultraviolet to the visible spectrum (with greater intensity in the ultraviolet and blue regions) (50). The characteristic blue glow of Cherenkov luminescence has been reported by astronauts in space and patients undergoing radiation therapy due to the stimulation of retinal pigments caused by the production of Cherenkov light, and it can have significant applications in the fields of stereotactic radiosurgery and brain radiation therapy (51).

The interaction between Cherenkov radiation and the GO-BSA-CTAB-PpIX nanostructure offers distinct advantages compared to conventional photodynamic therapy (PDT), particularly regarding efficiency and reactive oxygen species (ROS) generation. Conventional PDT relies on external light sources, such as lasers, to activate the photosensitizer, which limits the depth of light penetration and restricts the treatment to superficial or near-surface tumors. In contrast, Cherenkov radiation, generated internally during radiotherapy by high-energy particles, bypasses the limitation of light penetration, activating the photosensitizer deep within the tumor microenvironment. This synergistic approach allows simultaneous radiotherapy and photodynamic therapy, enhancing therapeutic efficacy.

The GO-BSA-CTAB-PpIX nanostructure is specifically designed to maximize the production of ROS upon activation by Cherenkov radiation. Including PpIX as the photosensitizer ensures efficient ROS generation due to its high quantum yield, while the GO component facilitates electron transfer, amplifying oxidative stress. Furthermore, the BSA coating improves biocompatibility and cellular uptake, while CTAB contributes to the stabilization and efficient delivery of the nanostructure. Together, these components optimize the interaction with Cherenkov radiation and enhance ROS production compared to conventional PDT systems.

Overall, integrating Cherenkov radiation with the GO-BSA-CTAB-PpIX nanostructure addresses key limitations of traditional PDT by enabling deeper tumor targeting, leveraging the synergistic effects of radiotherapy and PDT, and enhancing ROS generation. This innovative approach represents a promising avenue for overcoming challenges associated with conventional PDT and warrants further exploration in preclinical and clinical settings.

When the GBCP nanostructure is exposed to 6 MeV x-ray radiation and 200 cGy dose in aqueous medium (or tissue-equivalent medium), it is subjected to a massive influx of photons caused by the direct effect of Cherenkov radiation and the indirect effect of Cherenkov radiation (BSA fluorescence), which leads to Induced photodynamic effect is caused by X-ray Cherenkov radiation (RTCR-PDT). According to the results of cell studies and considering each of the hypotheses of pseudo-hormesis or induction of differentiation of cancer stem cells caused by the RTCR-PDT protocol, it can be concluded that the occurrence of Cherenkov radiation at the same time as radiation therapy can cause irreversible cell death on cervical cancer cells by GBCP nanostructure.

A) Size analysis of GBCP, B) zeta potential of GBCP, C/D) TEM and FE-SEM of GO, E/F) FE-SEM results of GBCP

GBCP: GO-BSA-CTAB-PpIX, GO: Graphene oxide nanostructure, BSA: Bovine serum albumin, PpIX: Protoporphyrin-9 , CTAB: Cetrimonium bromide

**Table 1:** Concentration ratios of the components of the GO-BSA-CTAB-PpIX nano complex

GO-BSA-CTAB-PpIX				Group number
PpIX (µg/ml)	BSA (µg/ml)	CTAB (µg/ml)	GO (µg/ml)	
40	1	12160	128	1
20	2	6080	64	2
10	3	3040	32	3
5	4	1520	16	4
2.5	5	760	8	5

A) Uv-Vis spectrophotometric analysis results of GO-BSA-CTAB-PpIX nanostructure at different times after synthesis (optical stability results). B) FTIR spectra of BSA, GO, GO-BSA-PpIX, and GO-BSA-CTAB-PpIX compounds

GBCP: GO-BSA-CTAB-PpIX, GO: Graphene oxide nanostructure, BSA: Bovine serum albumin, PpIX: Protoporphyrin-9 , CTAB: Cetrimonium bromide

A) Results of the spectrum of Cherenkov radiation in the presence of water and without it in the designed phantom. B) Differential average spectrum of Cherenkov radiation. Simultaneous examination of DPBF bleaching as a dosimetry probe for singlet oxygen and MB as a dosimetry probe for hydrogen peroxide under 6 MeV radiation in box mode with water (RTCR: Radiation + Cherenkov) and without water (RT: Radiation). In the conditions of C) Absence of GBCP hybrid nanostructure and D) Presence of GBCP hybrid nanostructure (GBCP: GO-BSA-CTAB-PpIX)

GBCP: GO-BSA-CTAB-PpIX, GO : Graphene Oxide Nanostructure, BSA: Bovine Serum Albumin, PpIX: Protoporphyrin-9 , CTAB: Cetrimonium bromide

**Table 2:** Checking the normality of the GBCP (Toxicity/Radiation) data with Kolmogorov-Smirnov test

GBCP-Toxicity					
0.1840		0.3090		0.1830	KS distance
>0.1000		0.0758		>0.1000	*P* value
Yes		Yes		Yes	Passed normality test (alpha=0.05)?
GBCP-Radiation					
0.2729	0.2402		0.2475		KS distance
0.0809	>0.1000		>0.1000		*P* value
Yes	Yes		Yes		Passed normality test (alpha=0.05)?

GBCP: GO-BSA-CTAB-PpIX, GO : Graphene oxide Nanostructure, BSA: Bovine serum albumin, PpIX: Protoporphyrin-9 , CTAB: Cetrimonium bromide

GBCP: GO-BSA-CTAB-PpIX, GO: Graphene oxide nanostructure, BSA: Bovine serum albumin, PpIX: Protoporphyrin-9 , CTAB: Cetrimonium bromide

GBCP: GO-BSA-CTAB-PpIX, GO: Graphene oxide nanostructure, BSA: Bovine serum albumin, PpIX: Protoporphyrin-9 , CTAB: Cetrimonium bromide

**Table 3:** The Comparison of therapeutic indicators of Cherenkov radiation Index (CRI), Radiation Index (RI), and Therapeutic Efficiency (TE)

72 hr	48 hr	24 hr	Indicator
73.8	2.29	16.6	CRI^*^
1.11	1.24	4.01	RI^*^
66.25	1.83	4.13	TE

GBCP: GO-BSA-CTAB-PpIX, GO: Graphene oxide nanostructure, BSA: Bovine serum albumin, PpIX: Protoporphyrin-9, CTAB: Cetrimonium bromid, RT: Radiation therapy, RTCR: Radiation therapy and Cherenkov radiation, PDT: Photodynamic therapy

GBCP: GO-BSA-CTAB-PpIX, GO: Graphene oxide nanostructure, BSA: Bovine serum albumin, PpIX: Protoporphyrin-9, CTAB: Cetrimonium bromide, RT: Radiation therapy, RTCR: Radiation therapy and Cherenkov radiation, PDT: Photodynamic therapy

## Declaration

In this article, it is important to clarify that artificial intelligence tools (Chat-GPT and Gemini) were utilized in select paragraphs solely to improve the clarity and conciseness of the writing. Nonetheless, all data and findings presented herein are the direct results of experimental work conducted in the laboratory.
